# Digital Twin Modeling of Flexible Perovskite Nano-Films with In-Situ Mechanical Microscopy Validation

**DOI:** 10.3390/nano13172388

**Published:** 2023-08-22

**Authors:** Melissa Ann Davis, Mehul Tank, Michelena O’Rourke, Matthew Wadsworth, Zhibin Yu, Rebekah Sweat

**Affiliations:** 1 High-Performance Materials Institute, FAMU-FSU College of Engineering, Tallahassee, FL 32310, USA; madavis@fsu.edu (M.A.D.); mtank@fsu.edu (M.T.); mwadsworth@fsu.edu (M.W.); 2 Notre Dame College of Engineering, Notre Dame, IN 46556, USA; morourk6@nd.edu

**Keywords:** flexible perovskite solar cells, mechanical deformation, finite element analysis, modeling, nano-cracking, in situ SEM

## Abstract

Flexible perovskite solar cells introduce opportunities for high throughput, high specific weight, and short energy payback time photovoltaics. However, they require additional investigation into their mechanical resiliency. This work investigates the mechanical properties and behaviors of perovskite thin films and builds a robust model for future research. A two-pronged approach was utilized. Perovskite thin films were flexed in a three-point bend mode with in-situ SEM. Novel insights into the perovskite mechanical behaviors with varying substrate layers were gained. Modeling and validation, the second prong, was completed with finite element analysis. Model coupons of the imaged perovskite architectures were built, with sensitivity analysis completed to provide mechanical property estimates. The results demonstrate that mechanical degradation of perovskite thin films on polyethylene terephthalate (PET) primarily presents as a crack in the grain boundaries between crystals. Perovskite thin films on Indium Tin Oxide (ITO) and PET primarily crack in a periodic pattern regardless of the placement of perovskite crystals.

## 1. Introduction

Perovskite solar cells have rapidly become a promising photovoltaic material. Its efficiency currently rivals silicon solar cells, the commercial leader, while its low cost and short energy payback time are distinct advantages [1,2]. Additionally, perovskite/silicon tandem solar cells have now demonstrated an efficiency of 33.2%, thus besting any other dual junction technologies, including III-Vs [1]. Flexible perovskite solar cells, one of the proven applications of perovskites, tout numerous advantages, from their pliable nature to their record high specific power, which is defined as the power-to-weight ratio [3,4]. In addition to the benefit of utilizing high-throughput manufacturing methods such as roll-to-roll printing, flexible perovskite solar cells expand the market due to their ability to be integrated into vehicles, drones, or backpacks.

Flexible perovskite devices have been reported in the literature as early as 2013 to have an efficiency of 2.62%, which has grown to a maximum certified efficiency of 23.8% [5,6]. However, flexible perovskite solar cells require additional research into mechanical properties and degradation behaviors. A thorough study is needed to understand the relationship between the perovskite grain geometry and the interconnecting layers with mechanical resiliency to bending.

Research into the mechanical properties of the perovskite material has begun, yet a sizable amount is still required. Mechanical investigations are roughly split into two fields: one of which focuses on mechanical stresses within the perovskite due to expansion and contraction, such as from thermal sources, while the second focuses on mechanical stresses due to physical forces on the material, such as a bending motion. The former field is arguably more investigated than the latter. Rolston et al. have reported work on determining the mechanical stability of rigid devices utilizing a double cantilever beam setup and nanoindentation [7,8]. Their work quantifies cohesion energy for various cation compositions on rigid substrates; however, its limitations are that their procedure does not concisely describe mechanical behaviors within the perovskite layer itself. Simulation research into this field has also been completed by Yang et al. Finite element analysis was completed on thermal stresses of bulk materials; however, insight was focused on optimizing interface layer selection to minimize Tresca equivalent stress [9]. In addition to these studies, we need an even more in-depth report on mechanical degradation methods, specifically on flexible devices.

The latter field, mechanical stresses of perovskite films due to bending, is noted in most reports of flexible perovskite devices. The commonly used method to report mechanical resiliency is to state the efficiency of devices before and after the cyclic bending of cells over a stated radius [10,11]. While this is a start in reporting on the mechanical resiliency of devices, it uses electrical data to describe mechanical performance, and we feel it is critical to instead utilize mechanical data to depict mechanical performance. This will allow researchers to view stresses and strains within the layers instead of viewing the devices as a unit, relating efficiency to mechanical resiliency. Tavakoli et al. provided one of the first reports of simulating mechanical stresses of perovskite films; their work utilizes a nanocone substrate to mitigate stresses from bending [12]. Du et al. more recently published simulation-based research on flexible perovskite solar cells with an emphasis on how electrical phenomena differ under a bending scenario [13]. Both papers placed their simulation coupons in a cantilever position. Extracting information from this setup is difficult, as the boundary conditions and forces in the model can distort the actual values of mechanical stresses. In this work, we place samples in a three-point bending scenario, which allows researchers to extract values of stresses of the impacted area, where the effect of boundary conditions on the edges is minimized.

We use a dual approach to report on this field through the utilization of finite element analysis along with novel SEM imaging methods to allow for realistic perovskite grain structure geometry. We aim to pinpoint the mechanisms of failure in a robust model that can be applied to any perovskite composition or architecture. Typical SEM imaging is constrained to the surface or cross-sectional area of one location, whereas, here, we render a 3D view of the entire device. In addition, finite element analysis allows for predictive cracking, which is verified with in situ SEM imaging under active bending. The ability to begin with a defect-free simulation coupon allows the results to be solely based on simulation instead of prerequisite defects. This approach also allows for discrete values for mechanical values of layers, such as Young’s modulus and tensile strength, including values for the perovskite crystal and their grain boundaries separately.

## 2. Materials and Methods

Materials: All materials were used as received: lead(II) iodide (AnhydroBeads 99.999%), dimethyl sulfoxide (DMSO) (anhydrous > 99.9%), y-butyrolactone (GBL) (>99%), and chlorobenzene (anhydrous 99.8%) from Sigma-Aldrich, methylammonium iodide (MAI) from One-Material, ITO coated PET from Delta-Technologies, and PET from McMaster-Car.

Film Fabrication: MAPbI3 solution was prepared by dissolving MAI with DMSO and GBL in a 7:3 ratio with a 1.2 M concentration. The resulting solution was added to PbI_2_ in a 1:1 molar ratio and stirred at 40 °C until PbI_2_ fully dissolved. PET and ITO-coated PET were subjected to a 5 min soak of Acetone and then IPA, followed by drying with compressed gas. Once dry, the substrates were treated with oxygen plasma for 5 min. A total of 100 µL of the MAPbI3 solution was deposited onto the desired substrates and subsequently spun at 1.5 k rpm/min for 4 s, followed by 3.5 krpm/min for 40 s. A total of 60 µL of chlorobenzene was dropped at 30 s, and the resulting film was annealed at 100 °C for 1 h.

Characterization: Perovskite-coated substrates of size 15 × 20 mm were imaged with the FEI Helios G4 UC SEM. The samples were placed into a Psylotech tensile frame. A slight prestress was added to ensure samples would bend upwards to image the perovskite in a convex orientation. The samples had 100 µm steps at a speed of 0.1 mm/s per step.

## 3. Results

The research consisted of two parts: in situ imaging of perovskite thin films and true geometry digitization of the perovskite bending model with experimental validation. As behavior validation was necessary for upgrading the simulation model, a focus on the in situ portion of the research will be discussed first.

### 3.1. In Situ SEM Mechanical Microscopy

**Figure 1:**
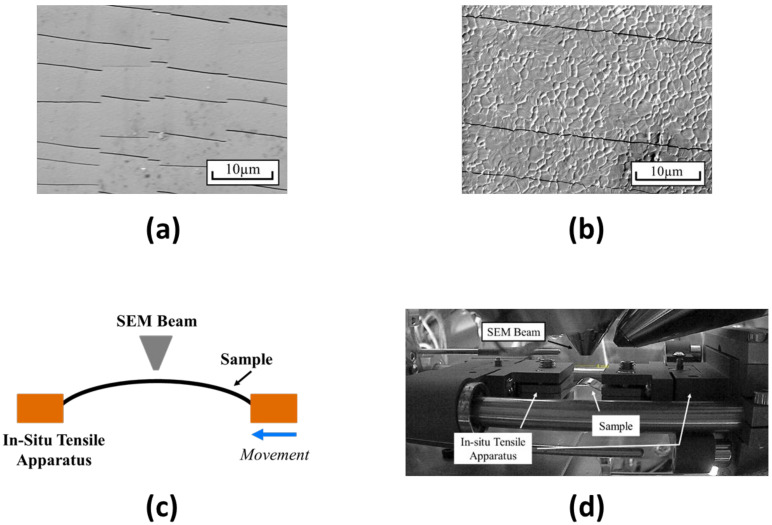
(**a**) SEM image of perovskite on ITO-coated PET after bending; (**b**) SEM image of ITO-coated PET after bending; (**c**) schematic depiction of in situ SEM setup; (**d**) image of in situ SEM setup.

Preliminary testing with traditional SEM was first completed before the use of the novel in situ SEM equipment. These tests were completed by imaging two stacks of materials: indium tin oxide, or ITO, coated polyethylene terephthalate, or PET, and perovskite on ITO-coated PET. The two stacks had imaging completed before and after extreme bending. These images were taken with a static and flat sample in the traditional manner of using SEM. Figure 1 shows the afterimages of the ITO-coated PET with and without the perovskite layer. It shows that cracking can be seen in straight and periodic lines. These lines appear perpendicularly to the bending radius or parallel to the edges of the sample that were brought together. In the image with the perovskite layer atop the ITO-coated PET, a similar cracking pattern was seen.

For the in situ SEM microscopy, two types of architectures of perovskite films were also tested. One of the architectures was perovskite on ITO-coated PET, while the other was perovskite on plain PET. The perovskite films had an MAPbI_3_ composition, as described in the experimental section.

After the deposition of gold via sputtering, in situ SEM imaging was completed on the two types of samples. Mechanical degradation of perovskite films was captured in real time due to the use of the Psylotech tensile frame. The samples were held in a tensile testing apparatus with the perovskite film facing toward the SEM beam. A slight prestress was applied to ensure that further displacement would ensure the perovskite would stress upwards towards the beam in a convex position. SEM images were collected of the samples as the in situ apparatus stepped inwards, reducing the radius of the samples that were stressed, thus increasing the chance for mechanical degradation. This setup can be seen in Figure 1.

ITO is one of the most commonly used transparent conducting electrodes for perovskite solar cells. It has excellent optical and electrical properties, but its mechanical properties are poor for flexible applications. The mechanical degradation behavior of ITO is buckling delamination [14,15]. Preliminary SEM images of ITO on PET after bending or folding the material demonstrate parallel cracks with moderately uniform and periodic spacing [16]. When samples of perovskite films on ITO and PET were tested in the in situ apparatus, a similar pattern was seen as well. The cracking behaviors of these samples demonstrate increasing cracks with a reducing radius. Cracks appear to form regardless of perovskite grain boundaries, with the understanding that grain boundaries viewed in this manner may demonstrate some inaccuracies.

**Figure 2:**
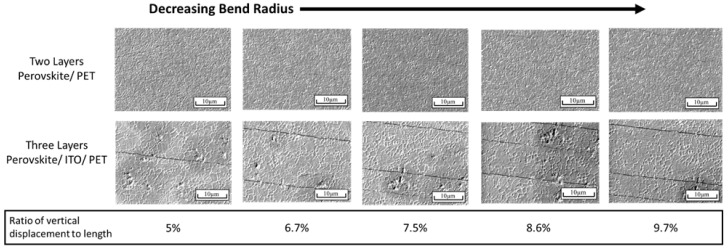
SEM images of perovskite samples with ITO-coated PET above the perovskite on PET below with decreasing bend radius from left to right, and the ratio of the displaced distance of three-point bend against the length of sample.

The perovskite films without an ITO inner layer were imaged in the same manner. The cracking behaviors of these films showed a distinct difference from the previously discussed films with ITO. Perovskite films on PET showed mechanical deformation that was primarily localized to the grain boundaries. The films demonstrated an expansion behavior in the grain boundaries as the bend radius decreased. The cracking or expansion was not seen in a straight line perpendicular to the reducing radius. It instead was disorganized and disjointed. Some cracking along crystals was apparent; however, cracking behaviors of perovskite on PET tended to primarily focus on the grain boundaries. A comparison of the two architectures with similar percentages of deflection is seen in Figure 2. A closer view of the two-layer SEM images can be found in the Supplementary Materials in Figure S1, as the cracks are difficult to see when compared to the three-layer images.

### 3.2. Digital Twin Research with Finite Element Analysis

Due to the insights into cracking behaviors of perovskite films on differing substrates during in situ SEM imaging with mechanical deformation, two new behaviors can be used as a comparison for their digital twin models. This research is a continuation of our previously published work that introduced a comprehensive look into a digital twin model of perovskite films [17]. As a continuation of this work, the same finite element analysis software, Simcenter MultiMech, was used. The model was a rectangular testing coupon with three layers subjected to a three-point bend. Boundary conditions restricting movement were assigned on the top edges of the sample, while a displacement was applied in the center bottom. One of the simulation coupons included three layers: PET, ITO, and perovskite. The layers were placed sequentially, with PET as the bottom layer, ITO in the middle, and the perovskite layer on top. A second simulation coupon included only two layers: PET and perovskite, with a similar architecture without the ITO layer.

The simulation coupon with three layers had a length of 10 µm, a width of 3 µm, and a depth of 2 µm. The coupon with two layers had the same length and width dimensions except for a slightly thinner depth of 1.9 µm due to the exclusion of the ITO layer. The thicknesses of the ITO and the perovskite layer closely matched that of their physical counterparts. ITO on PET commonly has a commercial thickness of 100 nm. The perovskite layer thickness does have a wider range in literature. A thickness of 400 nm was selected as it closely aligned with the thickness of the perovskite layers created experimentally. While the thickness of the perovskite layer may differ from other values reported in the literature, a consistent value was necessary to compare simulation results. In addition, the thickness of the layer can easily be adjusted for future research with the code.

The final layer, PET, does have a reduced thickness in the simulation coupons. The thickness of PET used experimentally has a value that is three orders of magnitude larger than the perovskite and ITO layers. Therefore, the thickness of PET was significantly reduced to 1.5 µm. This was selected to minimize computation time and focus on the upper layers. It would be illogical to model ITO or the perovskite layer without a base as it is required experimentally; however, mechanical degradation of PET is theorized to have minimal impact on the device stack. Reducing the thickness of the base polymer was also shown in the work of Tavakoli et al. [12].

The first author previously published a simulation method for modeling the bending of perovskite thin films; with this work, a true geometry of the materials is further realized [17]. Previously, the perovskite crystals were modeled with hexagonal shapes to represent their crystallinity. With this work, the perovskite crystals have been upgraded to represent a perovskite crystal that has been imaged physically.

The improved geometry of the new model was accomplished with custom-designed software programs. An algorithm designed in MATLAB first converted the captured SEM image from grayscale to binary to prepare the image for digitization. SEM images of perovskite crystals in a top-down orientation were selected as inputs to this process. The images were from samples on a flat or unbent substrate. 

With the resulting binary output of the perovskite crystal image, it is processed by the Harris–Stephens algorithm. The Harris–Stephens algorithm is used to graph a point at detected corners or places where pixels have a great contrast from their neighboring pixels. The resulting points were displayed with a custom graphical user interface or GUI. This GUI enabled the building of perovskite crystal boundaries through a selection of the points from the algorithm. Once all of the boundaries were selected, the GUI output the corresponding G-Code, which was the framework for the updated geometry coupon.

**Figure 3:**
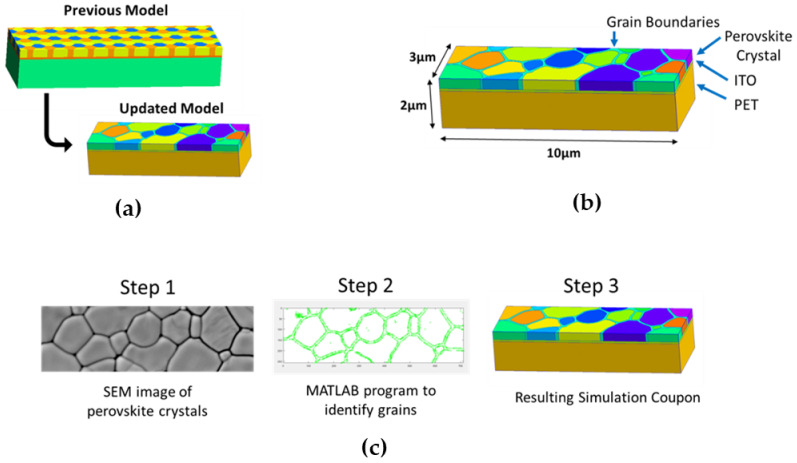
(**a**) Previous and updated model; (**b**) size and layers of updated simulation coupon; (**c**) process of digitizing the SEM image to resulting simulation coupon.

This process allowed for a fully digitized image from an SEM still. Utilization of a custom program allowed for highly accurate geometries, which would have taken significantly longer to attempt with lower accuracy. The upgrade in geometry added an additional entity, the perovskite grain boundary. This process is summarized in Figure 3. In addition, the digitization code and a video tutorial can be found in Supplementary Materials under Code S1 and Video S1 respectively.

With the creation of the updated model-building method, three simulation coupons were created. SEM images of perovskite crystals at varying locations on samples were selected. To ensure the simulation was robust as well as accurate, a larger simulation pool was utilized. Each of the three simulation coupons was created in the two architectures previously tested with in situ SEM: perovskite on ITO with PET and perovskite on PET. The six models were then subjected to a three-point bend in the software, and their resulting cracks and stresses were collected and analyzed. The framework for the simulation coupons, the geometry files, can be found in Supplementary Materials under Codes S2 and S3 which include a two and three layer code.

Mechanical property values were also adapted for the upgraded geometry. The foundation discovered in the previous research was used; however, the new geometry required further optimization of values. In addition, the upgraded geometry introduced a new entity of perovskite grain boundaries. Therefore, its mechanical values must be researched. It was previously discovered that tensile strength had the greatest control of model behavior. A parametric study was completed on the tensile strength of the perovskite grain as well as the interface strengths between the grain and crystal, as well as the interface strength between ITO and perovskite grain and perovskite crystal. The resulting values can be seen in Table 1.

Once parametric studies were completed, the simulation results were compared between samples as well as between the two architectures. With the knowledge gained from the in situ SEM, samples without the ITO layer are expected to crack in a linear fashion with moderate periodicity. This behavior was seen with the simulation; the cracks through the coupons with ITO-coated PET showed that the perovskite layer underwent significant cracking in the perovskite crystal compared to the coupons without ITO. An interesting phenomenon was seen with the video results, which can be viewed in the Supplementary Materials, where the first cracks in the perovskite layer on the samples with ITO occurred in the perovskite grain boundaries. The samples with ITO architectures first underwent perovskite layer cracking in the grain boundaries. However, significant cracking in the perovskite crystals quickly followed. A hypothesis of this behavior can be attributed either to the limits of simulation to exactly match physical results or due to the subtle nature of perovskite grain boundary cracking seen with SEM imaging.

**Figure 4:**
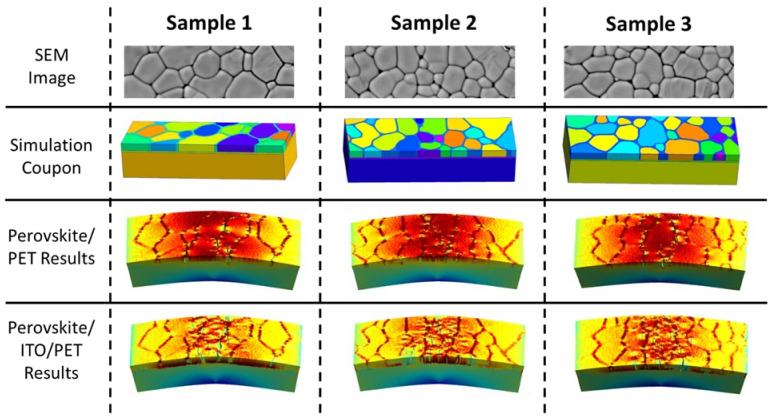
Comparison between Samples 1, 2, and 3 with SEM images, simulation coupons, and modeling results of two- and three-layer samples.

The second architecture, which lacked the ITO layer, was also analyzed. Although the architectures differed, the mechanical values for each remained the same. Cracking in the perovskite layer in simulation coupons without ITO was seen to be distinctly different from the simulation coupons with ITO. Cracking was primarily organized within perovskite grain boundaries, with minimal cracking found in the perovskite crystal itself. The perovskite crystals that exhibited cracks did so in the last few steps of the displacement. Cracking through the perovskite crystals also occurred in crystals close to the center point with a larger grain size than most other grains. This can be seen clearest in Sample 1. The yellow, centermost perovskite crystal in Sample 1 can be matched with the results of the simulation coupon without ITO to view the cracks through the crystal. The cracks that propagated through the crystal are close to the centermost point of the coupon, and the degraded crystal is also one of the largest in this simulation coupon nearest to the center. A summary of the comparisons between the three samples can be found in Figure 4. Videos from the digital twin model can be found in the Supplementary Materials in Videos S2–S7.

**Figure 5:**
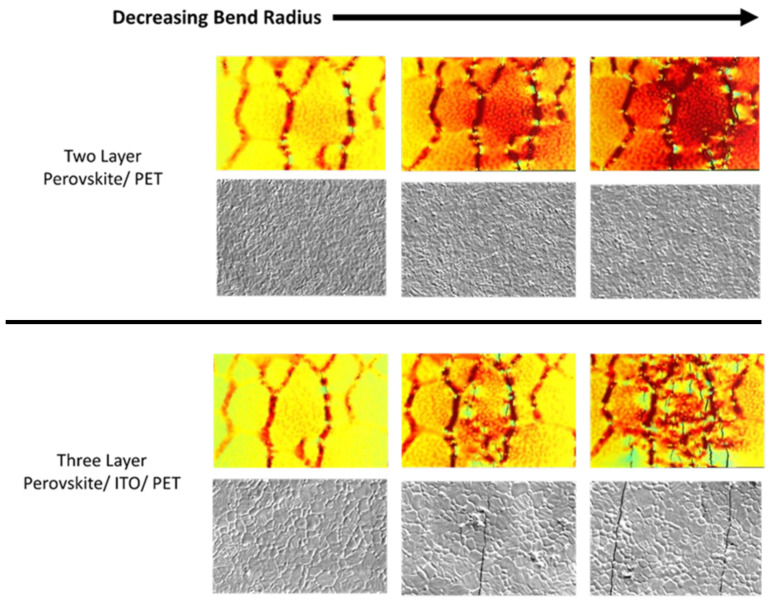
Comparison of two and three-layer simulation and SEM images with decreasing bend radius where simulation results are shown above SEM images of physical results.

Comparison between the physical samples and modeling results can be completed with some assumptions noted. As mentioned in previous work, the PET thickness in the model was decreased to simplify the model and reduce processing time significantly. Therefore, a direct comparison is illogical; however, adjustments to normalize bending ratios were completed. The bending ratio used for the comparison is the ratio between the vertical displacement of the centermost point in the three-point bend to the length of the sample. Adjustments were necessary due to the difference in thickness, as a thicker PET layer subjects the perovskite and other layers to a further vertical displacement than a thinner PET layer. As such, the graphic below should be viewed as an estimation. Despite the estimation, there is a clear connection of mechanical behavior between both research mediums. Cracking in the two-layer architecture demonstrates a tendency to crack in the grain boundaries in both the simulation and physical imaging, which differs from the behavior seen in the three-layer architecture, where a significant amount of the cracking occurs in the perovskite crystals. These behaviors can be seen in Figure 5 as a side-by-side comparison between simulation stills and SEM images.

## 4. Discussion

With the dual research approach of simulation and experimentation, a robust method to predict the mechanical deformation of perovskite films has been proven. While this model used MAPbI_3_ perovskite, additional formulations can be added in future work. Perovskite solar cells with record efficiencies use upwards of three cations and two halides. This model can rapidly test varying compositions to determine mechanical properties with the deposition of a singular layer. This allows for the ability to avoid optimization of the layers beneath the perovskite, such as a hole or electron transport layer.

In addition, with this model, future research can be conducted in two directions. With knowledge of mechanical behaviors, the system proposed can digitize films to run sensitivity analyses to determine accurate mechanical properties. A further direction is to start with known mechanical properties to then simulate resulting mechanical behaviors. The proposed research can also determine differences in the mechanical behavior of perovskite films through changing perovskite crystal geometries.

This research provides a new tool to determine accurate mechanical properties and behaviors of perovskite or other crystalline films. A valuable path for further research would be to link mechanical degradation with the efficiencies of full devices. It is reasonable to assume that there would be a threshold of allowable mechanical degradation before the performance begins to suffer. Future research can build layers upon the perovskite to more accurately model a full device stack.

This research demonstrates a novel method to accurately model the true geometry of perovskite films under a three-point-bend deformation. It also provides in situ imaging of perovskite films in a viewpoint commonly underreported due to novel apparatus.

## Figures and Tables

**Table 1:** Young’s modulus, Poisson’s ratio, and tensile strength from literature with references (* denotes estimated values from sensitivity analysis).

Values	PET	PET/ITO Interface	ITO	ITO/Perovskite Crystal Interface	ITO/Perovskite Grain Interface	$\mathrm{Perovskite}\;(\mathrm{MAPb}I_{3})$ **Crystal**	Perovskite Crystal/Grain Interface	Perovskite Grain Boundary
*Young’s Modulus (GPa)*	2.52 [18]		116 [19]			8.5 +		8.5 +
*Poisson’s Ratio*	0.405 [18]		0.35 [19]			0.33 [20,21,22]		0.33 [20,21,22]
*Tensile Strength (MPa)*	150 [17]	50	375 [23]	150 *	150 *	426 [24]	200 *	200 *

## Data Availability

The coding for this research can be found in Supplementary Materials.

## Supplementary Materials

The following supporting information can be downloaded at: https://www.mdpi.com/article/10.3390/nano13172388/s1, Figure S1: Zoomed-In SEM; Video S1: Matlab Code Tutorial, Video S2: Sample 1_2 Layer, Video S3: Sample 1_3 Layer, Video S4: Sample 2_2 Layer, Video S5: Sample 2_3 Layer, Video S6: Sample 3_2 Layer, Video S7: Sample 3_3 Layer; Code S1: Digitalization code (matlab), Code S2: Two Layer Geometry (geo file), Code S3: Three Layer Geometry (geo file).
