# Supplementary material for: Digital Twin Modeling of Flexible Perovskite Nano-Films with In-Situ Mechanical Microscopy Validation
Source: Nanomaterials (Basel). 2023 Aug 22;13(17):2388. doi: 10.3390/nano13172388 (PMC10490042; doi:10.3390/nano13172388)
Supplement: Supplementary file 1 [file nanomaterials-13-02388-s001.zip › Zoomed-In SEM.pdf]

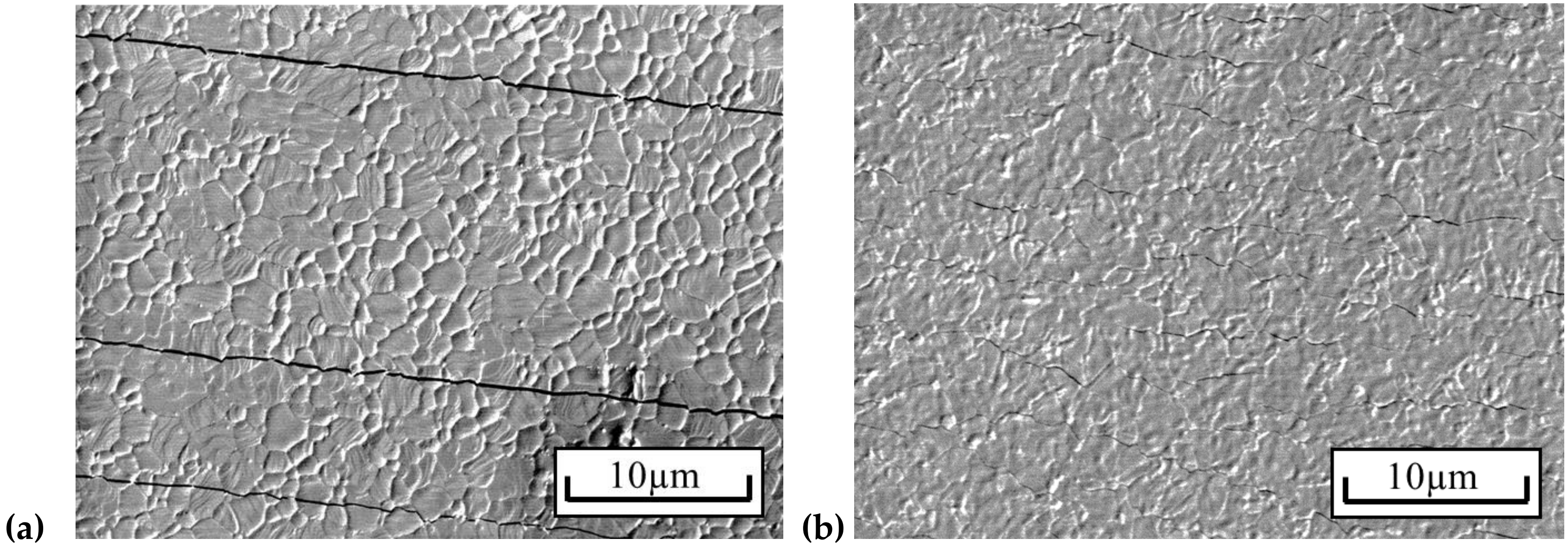

Supplementary Figure S1. Zoomed-in SEM image of (a) perovskite on ITO-coated PET and (b) perovskite on PET during in-situ imaging at the same displacement of 9.7% (which is a ratio of vertical displacement to length)
